# Retinal image synthesis from multiple-landmarks input with generative adversarial networks

**DOI:** 10.1186/s12938-019-0682-x

**Published:** 2019-05-21

**Authors:** Zekuan Yu, Qing Xiang, Jiahao Meng, Caixia Kou, Qiushi Ren, Yanye Lu

**Affiliations:** 1 Department of Biomedical Engineering, College of Engineering, Peking University, Beijing, 100871 China; 2 Beijing University of Posts and Telecommunications, Beijing, 100876 China; 3 Pattern Recognition Lab, Department of Computer Science, Friedrich-Alexander-University Erlangen-Nuremberg, 91058 Erlangen, Germany

**Keywords:** Retinal image synthesis, Generative adversarial networks, Multiple landmarks

## Abstract

**Background:**

Medical datasets, especially medical images, are often imbalanced due to the different incidences of various diseases. To address this problem, many methods have been proposed to synthesize medical images using generative adversarial networks (GANs) to enlarge training datasets for facilitating medical image analysis. For instance, conventional methods such as image-to-image translation techniques are used to synthesize fundus images with their respective vessel trees in the field of fundus image.

**Methods:**

In order to improve the image quality and details of the synthetic images, three key aspects of the pipeline are mainly elaborated: the input mask, architecture of GANs, and the resolution of paired images. We propose a new preprocessing pipeline named multiple-channels-multiple-landmarks (MCML), aiming to synthesize color fundus images from a combination of vessel tree, optic disc, and optic cup images. We compared both single vessel mask input and MCML mask input on two public fundus image datasets (DRIVE and DRISHTI-GS) with different kinds of Pix2pix and Cycle-GAN architectures. A new Pix2pix structure with ResU-net generator is also designed, which has been compared with the other models.

**Results and conclusion:**

As shown in the results, the proposed MCML method outperforms the single vessel-based methods for each architecture of GANs. Furthermore, we find that our Pix2pix model with ResU-net generator achieves superior PSNR and SSIM performance than the other GANs. High-resolution paired images are also beneficial for improving the performance of each GAN in this work. Finally, a Pix2pix network with ResU-net generator using MCML and high-resolution paired images are able to generate good and realistic fundus images in this work, indicating that our MCML method has great potential in the field of glaucoma computer-aided diagnosis based on fundus image.

## Background

Computer-aided diagnosis (CAD) systems benefit physicians in reducing workload and improving diagnostic accuracy for medical examination. Deep learning methods, especially convolutional neural networks (CNNs), have achieved great success in many computer vision tasks such as image classification [1], detection [2], and segmentation [3]. With regard to medical applications, there are many breakthroughs [4–6] using deep learning-based methods. In order to train a successful CAD model for medical image analysis, researchers need to collect large amounts of training data. Additionally, imbalanced medical image datasets and shortage of good experts annotating data are two main problems for improving the performance of the model. In order to address these problems, some methods have been proposed to generate artificial medical images to improve the performance of CAD systems [7–9]. Synthetic images can be used to enlarge the training sets and therefore improve the performance of the segmentation and classification tasks.

Generative adversarial networks (GANs) [10] are a family of unsupervised machine learning algorithms that have demonstrated their merits through generating synthetic images and solving image-to-image translation problems in natural image domain [11]. Typically, GANs are implemented by a system of two neural networks competing with each other in a two-player zero-sum game: a discriminator network and a generator network. The generator produces candidates mapped from a latent variable to an objective data distribution, while the discriminator discriminates between the true data distribution and the candidates. Lots of new GAN structures have been developed, such as W-GAN [12], Info-GAN [13], DCGAN [14], CGAN [15], Pix2pix [11], Cycle-GAN [16], and most of them have achieved good performance in image-to-image translation tasks.

Recent literature regarding medical image synthesis has presented good results in different medical imaging modalities. Salehinejad et al. [7] proposed to generate X-rays images for chest pathology classification by convolutional generative adversarial network (DCGAN) [14]. They have shown that artificial data can improve the classification accuracy better than both imbalanced and balanced real datasets. A method named MelanaGANs was proposed by Bissoto et al. [8], which can synthesize high-resolution dermoscopic images with melanoma lesions. This method has been further demonstrated that synthetic images can make contributions to solve the problem of class imbalance and improve classification accuracy. Hou et al. [17] proposed a GAN-based method to synthesize images in various strategies. They found that synthetic images of cancer cells improve the segmentation results, and reduce the error of cell nucleus segmentation by 6 to 9%. Hu et al. [18] presented an approach to simulate fetal ultrasound images based on conditional GANs, generating realistic ultrasound images at target 3D spatial locations. Such approach is valuable in obstetric examination. Shin et al. [9] proposed an algorithm to produce synthetic abnormal brain tumor MRI images from their corresponding segmentation masks with GAN. They have demonstrated the improved performance on tumor segmentation by leveraging synthetic images.

Fundus imaging is a basic check-up process in ophthalmology, which provides useful information to facilitate ophthalmologist in diagnosing different ocular diseases at early stages [19]. In the task of color fundus image generation, many methods have been developed [20–22]. Among them, Costa et al. [20] developed a method that learned to map from vessel tree to retinal image based on Pix2pix model [11], which utilizes similar techniques to U-net [23], preserving global structural information during the data generation process. Zhao et al. [21] synthesized fundus images combined from vessel tree ground truth based on Pix2pix framework, and the results showed that synthetic fundus images combined with real images outperform only real fundus images input method in fundus vessel segmentation tasks. All works proposed end-to-end adversarial retinal image synthesis pipelines, generating new vessel trees to synthesize retinal images with reasonable performance. However, important landmarks of eye, such as vessel parts, optic discs, and optic cups, have not been taken into account while synthesizing retinal images. The regions of optic disc and optic cup in artificial fundus image are blurry and unsmooth, resulting in potential issues for further applications. For instance, such as in analyzing glaucoma fundus image tasks, the main lesion part is the changes of optic disc and optic cup [24]; however, the regions of optic disc and cup in generated fundus image are blurry using the prior synthesis method, giving limited help to computer-aided diagnosis.

To address these shortcomings of the previous methods, we propose a novel multiple-channels-multiple-landmarks method as a new preprocess pipeline to synthesize color fundus images from a combination of vessel, optic disc, and optic cup through image segmentation. The image quality of the proposed MCML method is quantitatively evaluated for comparison studies with the state-of-the-art GANs-based synthetic methods such as Pix2pix [11] and Cycle-GAN [16]. Furthermore, the optimization of the above-mentioned generators (i.e., MCML-GANs, Pix2pix, and Cycle-GAN) has been investigated to get better synthetic image quality as well. The remainder of this paper is as follows: in “Methods” section, we introduce the GANs with different structures and the datasets we used in this work; in “Experimental evaluation” section, we elaborate on our proposed method in detail, and analyze the experimental results; in “Discussion” section we provide further discussion; finally, our conclusion is given in “Conclusions” section.

## Methods

### Flowchart of our approach

**Fig. 1:**
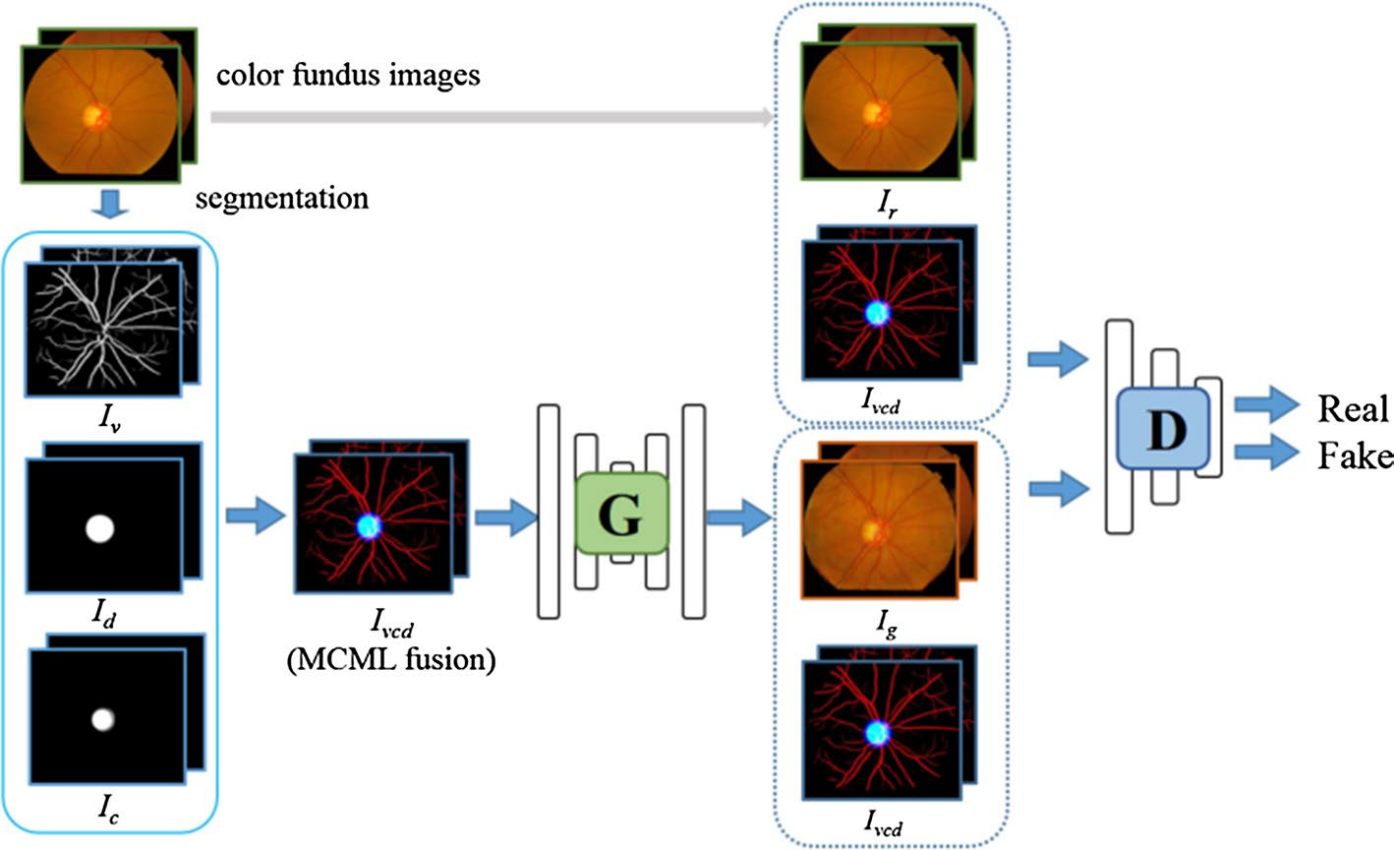
Overview of our approach. Color fundus image synthesis by multiple landmarks

An overview of the architecture is sketched in Fig. 1. As shown in the flowchart, the MCML-GANs method uses a combination of three channels: vessel (*I*_v_), optic cup (*I*_c_), and optic disc (*I*_d_). Prior works [20–22] only use vessel. Our goal is to learn a mapping function *I*_g_ = * G*(*I*_v_, *I*_c_, *I*_d_) between multiple landmarks (*I*_vcd_), synthesized from the three channels *I*_v_, *I*_c_, and *I*_d_, and corresponding real fundus image *I*_r_. The data distribution of the input samples is denoted as *I*_vcd_ ~ * P*_data_(*I*_vcd_) and paired real fundus images *I*_r_  ~  *P*_data_(*I*_r_), respectively.

### Adversarial translation from MCML-GAN to retinal images

In this work, the multiple-channels-multiple-landmarks (MCML) method is to map *I*_r_ ~  *P*_data_(*I*_r_*|I*_vcd_) given by *I*_vcd_. The combination of the three channels facilitates the generator adjusting the proportion of each structure automatically, avoiding setting weight hyperparameters for each structure. Then, a color fundus image is constructed by optimizing the latent parameters $\theta_{G}$ of a generator neural network *G*(*I*_vcd_). In the end, an independent unit *I*_vcd_ is mapped to the color fundus image.

In a zero-sum minimax optimization framework described in GANs [9], the generator is optimized through a discriminator neural network *D*(*I*_vcd_, *I*_r_) with a latent parameters $\theta_{D}$, which outputs a scalar likelihood classifying the input as true or false, i.e., real or fake color fundus image using multiple landmarks *I*_vcd_.

Therefore, the overall objective is to minimize min–max loss function, which is defined as

1 $$L_{\text{adv}} \left( {G,\;D} \right) = {\mathbb{E}}_{{I_{\text{vcd}} ,\;I_{\text{r}} \sim P_{\text{data}} (I_{\text{vcd}} ,\;I_{\text{r}} )}} \left[ {\log \;D\left( {I_{\text{vcd}} ,\;I_{\text{r}} } \right)} \right]\, +\, {\mathbb{E}}_{{I_{\text{v}} \sim P_{\text{data}} (I_{\text{v}} )}} \left[ {\log \left( {1 - D\left( {I_{\text{vcd}} ,\;G\left( {I_{\text{vcd}} } \right)} \right)} \right)} \right].$$

This is achieved by jointly optimizing the cost functions of both the discriminator and the generator, *L*(*G*) and *L*(*D*):

2 $$L\left( G \right) = {\mathbb{E}}_{{I_{\text{v}} \sim P_{\text{data}} \left( {I_{\text{v}} } \right)}} \left[ {\log \left( {1 - D\left( {I_{\text{vcd}} ,\;G\left( {I_{\text{vcd}} } \right)} \right)} \right)} \right],$$

3 $$L\left( D \right) = {\mathbb{E}}_{{I_{\text{vcd}} ,\,I_{\text{r}} \sim P_{\text{data}} \left( {I_{\text{vcd}} ,\;I_{\text{r}} } \right)}} \left[ {\log \;D\left( {I_{\text{vcd}} ,\;I_{\text{r}} } \right)} \right] + {\mathbb{E}}_{{I_{\text{r}} \sim P_{\text{data}} \left( {I_{\text{r}} } \right)}} \left[ {\log \left( {1 - D\left( {I_{\text{vcd}} ,\;G\left( {I_{\text{vcd}} } \right)} \right)} \right)} \right],$$

where ${\mathbb{E}}$ is a statistical expectation. Using sample pairs from data distribution (vcd, r) ~ *P*_data_(vcd, r), parameters $\theta_{G}$ and $\theta_{D}$ are, respectively, updated in each iteration to decrease the values of the *L*(*G*) and *L*(*D*) cost functions. Conceptually, the optimization of *L*(*G*) aims to enable *G*(*I*_vcd_) generating samples that the discriminator classifies as true images. Simultaneously, *L*(*D*) is optimized, in an adversarial manner, to correctly classify the images *G*(*I*_vcd_) produced by the generator and samples from the training dataset *I*_r_. Once a convergence condition was fulfilled, the generator is expected to generate samples from labels to color fundus images.

### Network architecture

Two types of GANs were implemented in this work for comparison studies. One is the Pix2pix [11] model that learns a generator from an image A to an image B. In order to produce more clarity and sharper results, we have referenced the recent improvements from [11, 25] as well, which propose to combine adversarial losses with a global L1 loss. The loss function can be optimized as

4 $$L(G,D) = L_{\text{adv}} (G,D) + \lambda {\mathbb{E}}_{{I_{\text{vcd}} ,\;I_{\text{r}} \sim P_{\text{data}} }} \left( {\left\| {I_{\text{r}} - G\left( {I_{\text{vcd}} ,\;I_{\text{r}} } \right)} \right\|_{1} } \right),$$

where *λ* balances the contribution of the two losses, and *λ* in this work is set as 0.5. The goal of the learning process is to find an equilibrium of Eq. 4. The L1 loss controls low-frequency information in the images generated by *G* in order to produce globally consistent results, whereas the adversarial loss promotes sharp results.

The other model is Cycle-GAN [16], which is good at realizing image-to-image translation tasks by unpaired datasets. The goal is to learn two generators *G*_1_ and *G*_2_ from image A to B and image B to A, respectively. The learning of two discriminators is to discriminate whether the picture is real or fake.

The training process is making *A*′ = *G*_2_(*G*_1_(*A*)) ≈ *A* and *B*′ = *G*_1_(*G*_2_(*B*)) ≈ *B*. It combines the adversarial loss with a global L1 loss between a cycle fake image B′ and the real image B. Thus, the loss function is

5 $$L(G_{1} ,G_{2} ,D_{1} ,D_{2} ) = L_{\text{GAN}} \left( {G_{1} ,D_{2} ,A,B} \right) \, + L_{\text{GAN}} \left( {G_{2} ,D_{1} ,B,A} \right) + \lambda L_{\text{cyc}} \left( {G_{1} ,G_{2} } \right),$$

where

6 $$L_{\text{GAN}} (G_{1} ,D_{2} ,A,B) \, = {\mathbb{E}}_{{b \sim P_{\text{data}} \left( b \right)}} \left[ {\log \;D_{2} \left( b \right)} \right] + {\mathbb{E}}_{{a \sim P_{\text{data}} \left( a \right)}} \left[ {\log \;1 - \;D_{2} \left( {G_{1} \left( a \right)} \right)} \right],$$

7 $$L_{\text{GAN}} (G_{ 2} ,D_{ 1} ,B,A) = {\mathbb{E}}_{{a \sim P_{\text{data}} \left( a \right)}} \left[ {\log \;D_{1} \left( a \right)} \right] + {\mathbb{E}}_{{b \sim P_{\text{data}} \left( b \right)}} \left[ {\log \;1 - D_{1} \left( {G_{2} \left( b \right)} \right)} \right],$$

and

8 $$L_{\text{cyc}} (G_{1} ,G_{2} ) = {\mathbb{E}}_{{a \sim P_{\text{data}} \left( a \right)}} \left[ {\left\| {G_{2} \left( {G_{1} \left( a \right)} \right) - a} \right\|_{1} } \right] + {\mathbb{E}}_{{b \sim P_{\text{data}} \left( b \right)}} \left[ {\left\| {G_{1} \left( {G_{2} \left( b \right)} \right) - b} \right\|_{1} } \right],$$

where *λ* controls the relative importance of the two objectives. Three types of generators were implemented for the GANs in this work.

1. U-net [23]: the U-net architecture does not have any fully connected layers, which are replaced by upsampling operators that are added skip connections between each convolutional layer. An overview of this process is shown in Fig. 2a, where the three colorful lines (red, green, and blue) represent the multiple landmarks inputs. Each blue box corresponds to a multi-channel feature map, and it includes a convolutional layer, a batch-normalization layer, and a ReLU activation. The number of channels is denoted on top of the box. The *x*–*y* size is provided at the lower left edge of the box. White boxes represent copied feature maps and green boxes represent residual block. Each residual block contains two convolution layers, two batch-normal layers and two ReLU activations. The arrows denote the different operations.

**Fig. 2:**
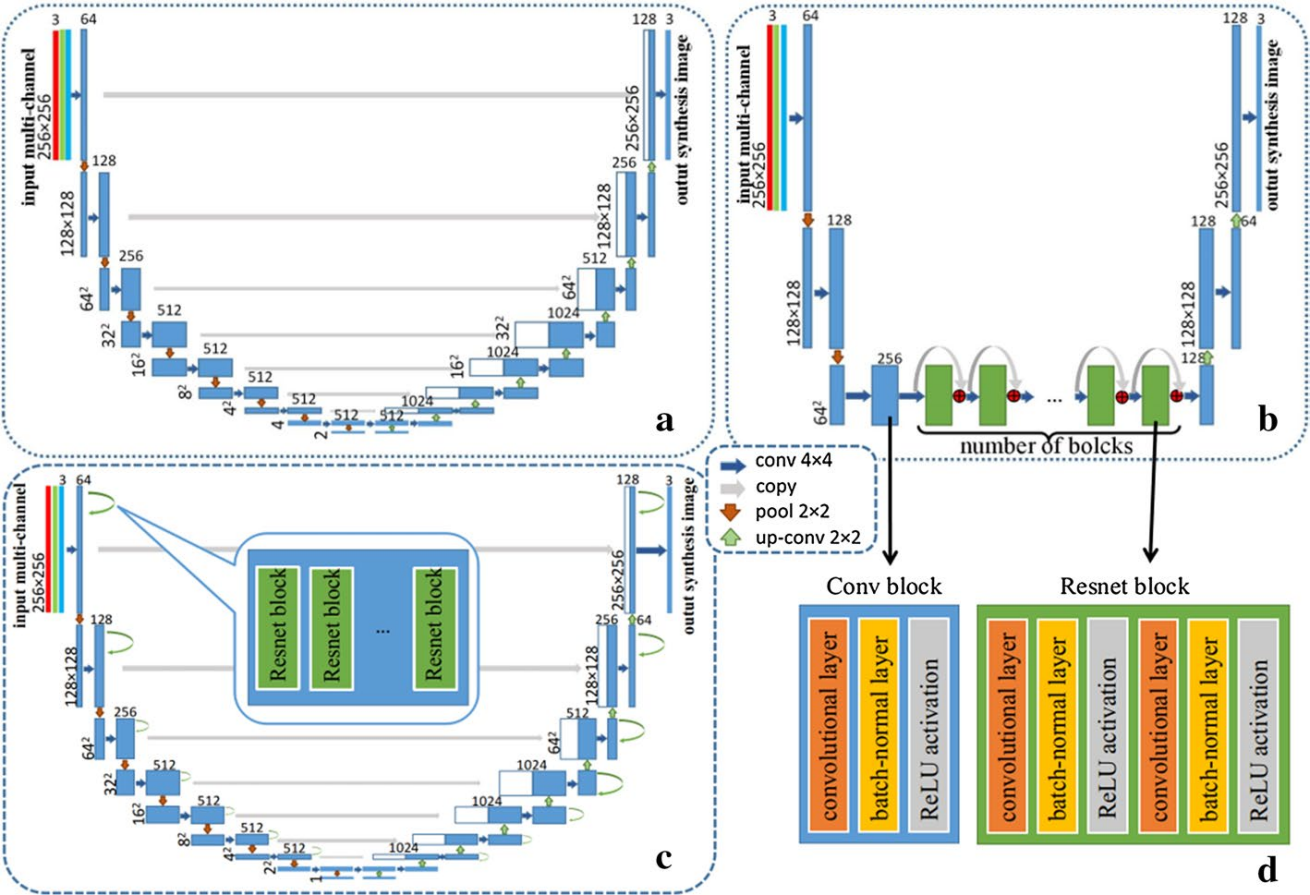
Generator network. **a** U-net. **b** ResNet. **c** ResU-net. **d** Details of blocks
2. Residual networks (ResNets) [26]: ResNets have demonstrated significant performance across many benchmarks in the computer vision field. The deep residual networks include a set of residual blocks, where information is propagated through a shortcut connection that bypasses the nonlinear layers with an identity mapping. A typical residual block consists of convolutional layers, rectified Liner Unit (ReLU) layers, and batch-normalization layers. Each residual block can be expressed as
  9 $$x_{l + 1} = x_{l} + F\left( {x_{\text{t}} ,\;W_{\text{t}} } \right),$$
  where *x*_*l*_, *x*_*l*+1_, and *W*_t_ are the input, output, and the convolutional weights of the *l*th residual block, respectively, and *F*(·) denotes the residual function corresponding to the *l*th unit. In the ResNets, several residual blocks were embedded into the fully convolutional network architecture to solve the bottleneck problem. The architecture is shown in Fig. 2b. A 6 block model and 9 block model were implemented in our experiment.
3. ResU-net: In order to take advantage of both U-net and ResNet, we have integrated residual blocks with a U-net architecture. The Integrated network we proposed is similar to ResU-net in Zhang et al. works [27], which has achieved better segmentation results in remote sensing images. ResU-net, which can better capture finer-scale details, still consists of a fine-to-coarse down-sampling path and a coarse-to-fine upsampling path with shortcut connections. For every two convolutional layers at the same resolution level in U-net, other network parameters are the same as the original U-net. The details of each block are shown in Fig. 2d. Similarly, we have also tested the 1, 2, and 3 residual block models for parameter tuning.

**Fig. 3:**
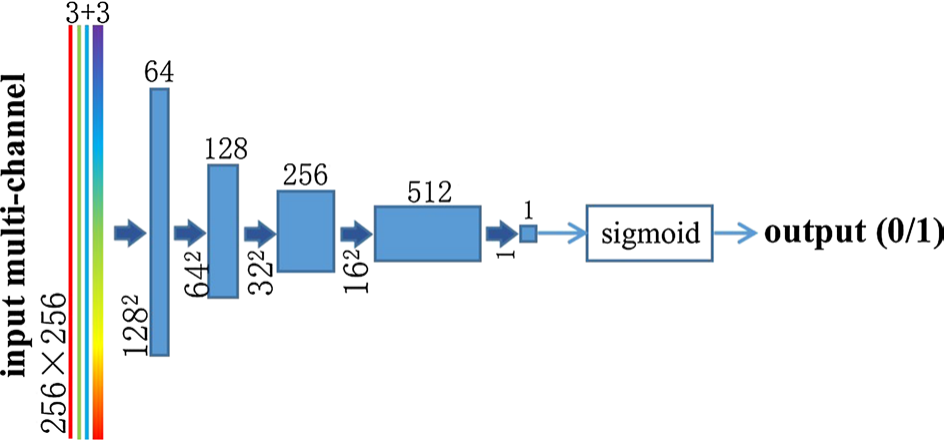
Discriminator network

The overview of the discriminator network is shown in Fig. 3, where the three red, green, and blue lines represent the multiple landmarks inputs, and colorful thick line represents the real fundus image. Each blue box corresponds to a multiple landmarks map, and it includes a convolutional layer, a batch-normal layer, and a ReLU activation. It end up with a sigmoid activation.

In this work, Single Vessel method and our MCML method are implemented by means of two different kinds of GANs:

1. Pix2pix: a GAN with different generators. In this work, U-net, ResNet with 6 residual blocks (ResNet-6), and ResNet with 9 residual blocks (ResNet-9) are used as the generators of Pix2pix.
2. Cycle-GAN: a GAN uses U-net, ResNet-6, and ResNet-9 as generators.

All the experiments were implemented by means of Python 3.6 and PyTorch on a NVIDIA 1080Ti GPU. During the training process, Adam [28] was used with a momentum parameter *l* = 0.5 and batch normalization. The generators were trained with an initial learning rate 0.0002 and a MSE loss function was used to initialize *G*. Since our training dataset is not very large, we trained the generators without using dropout [29]. All the experiments were trained using 200 update epochs.

### Datasets

In this work, two public datasets that provide fundus images are used for feasibility studies and comparison studies. DRISHTI-GS [30] is a comprehensive dataset of 101 retinal images that include 70 glaucomatous eyes and 31 normal eyes. All the images have the manual segmentations of optic disc and optic cup annotated by four experts. Since the DRISHTI-GS dataset does not contain manual vessel segmentations, a U-net-based vessel segmentation method [23], which was trained on another public dataset DRIVE [31], was used to segment vessel networks from DRISHTI-GS to provide the vessel ground truth for the dataset. Forty training images and ten testing images were randomly picked. All the images were resized from 286 × 286 resolution to 256 × 256.

The DRIVE dataset has 40 images captured in digital form from a Canon CR5 nonmydriatic 3CCD camera at 45 field of view. The vessel in the images was manually segmented in the dataset. We implement a similar segmentation method [32] to obtain the optic disc in the images. Fifteen training images and five testing images were randomly picked. All the images were resized from 565 × 584 resolution to 512 × 512.

### Evaluation metrics

In the prior works, Costa et al. [20] employed Image Structure Clustering (ISC) metric [33], which is a no-reference quality metric to evaluate the synthetic image quality. In [21, 22], all evaluate their synthetic fundus images by comparing the segmentation accuracy results between real and synthetic images. The accuracy measures the extent to which the synthetic image matches with the reality. In the other works, there were not uniform evaluation indexes with different vessel segmentation methods. In this work, we take optic disc and optic cup into consideration to synthesize more realistic images and have achieved better image quality. In order to objectively demonstrate the superiority of the proposed method, all the experiments were quantitatively evaluated using the structural similarity (SSIM) index [34] and the peak signal-to-noise ratio (PSNR). The SSIM index measures the similarity of structural information in two images, where 0 indicates no similarity and 1 indicates total positive similarity. PSNR measures image distortion and noise level between two images, a higher PSNR value indicates a higher image quality. SSIM are usually used in medical image prediction tasks [35–37] and larger SSIM index means that the synthetic fundus image is more close to the real one.

## Experimental evaluation

### Experimental results 

#### Subjective visual quality evaluation

**Fig. 4:**
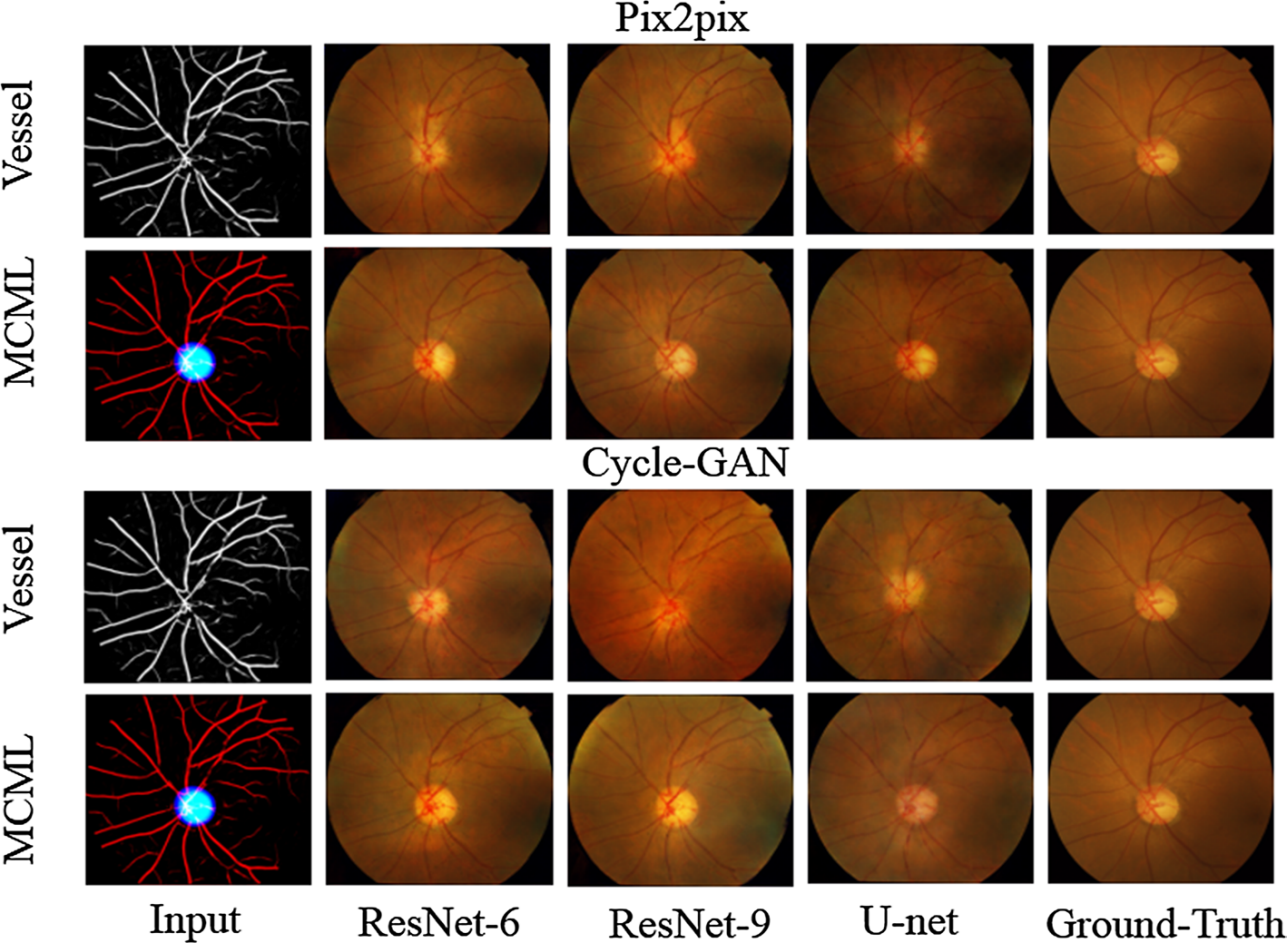
Exemplar synthetic images generated by our MCML-GAN method and only vessel input method. Pix2pix and Cycle-GAN with different architectures of generators were applied with these two input methods

Figure 4 shows the results of applying Single Vessel and MCML with GANs on DRISHTI-GS dataset. The MCML method has achieved better human visual perception than Single Vessel across each kind of GANs. As shown in Fig. 4, the optic cup parts of the synthetic images using Single Vessel are blurry and irregular, resulting in difficulty in observing the boundary of the optic cup. Compared to the single vessel results, the regions of the optic disc and optic cup generated by MCML are brighter and smoother. We can observe that the fundus images generated by our MCML method preserve not only the vessel vascular shape, but also the shape of the optic disc and optic cup. It is worth mentioning that, the details of shape, color, and texture in the region of the optic cup and optic disc are more similar to the original retinal images.

In order to further prove the superiority of the proposed method, we quantitatively validated our method using single-structure and MCML with different generative networks. As a result, it showed that our method is significantly better than the results of single vessel input method on all of generative networks, and the generated color fundus images show extraordinary results.

#### Quantitative evaluation

Table 1 presents the performance on average SSIM and PSNR across each method. It can be seen that the MCML method demonstrates superior performance in most cases compared to Single Vessel, indicating that MCML works better along with the GANs in this work. It is observed that Pix2pix has better performance than Cycle-GAN under the same generators, in which Pix2pix with ResNet-9 has achieved the best PSNR, and Pix2pix with U-net has achieved the best SSIM.

**Table 1:** Comparison of SSIM and PSNR on DRISHTI-GS dataset with different GAN models

Methods	SSIM	PSNR
Pix2pix		
ResNet-6 vessel	0.8983	23.8254
ResNet-6 MCML	0.8968	24.0820
ResNet-9 vessel	0.8914	23.5531
ResNet-9 MCML	0.9079	*25.3665*
U-net vessel	0.8956	23.4331
U-net MCML	*0.9117*	24.5544
Cycle-GAN		
ResNet-6 vessel	0.8770	22.4830
ResNet-6 MCML	0.9019	23.8092
ResNet-9 vessel	0.8774	21.9402
ResNet-9 MCML	0.8907	23.1031
U-net vessel	0.8984	22.7353
U-net MCML	0.8877	23.0110

Italic values are the best results

**Fig. 5:**
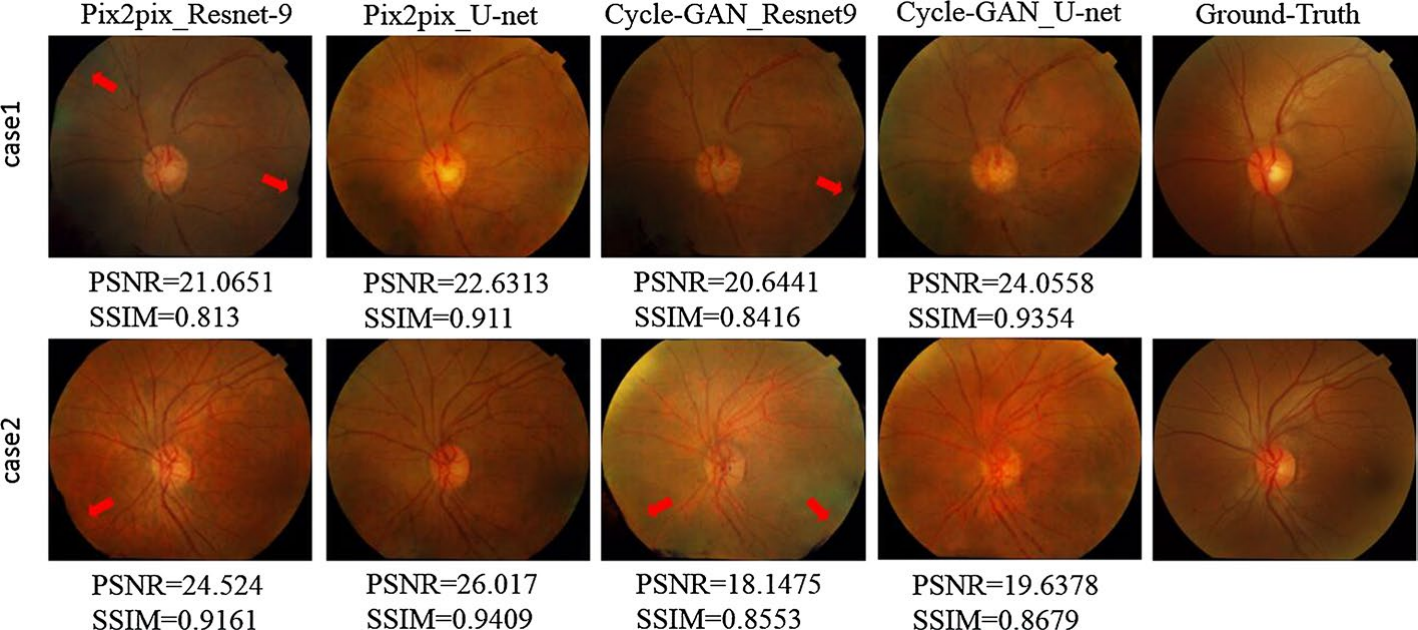
Comparison with different generator architectures based on MCML method

**Fig. 6:**
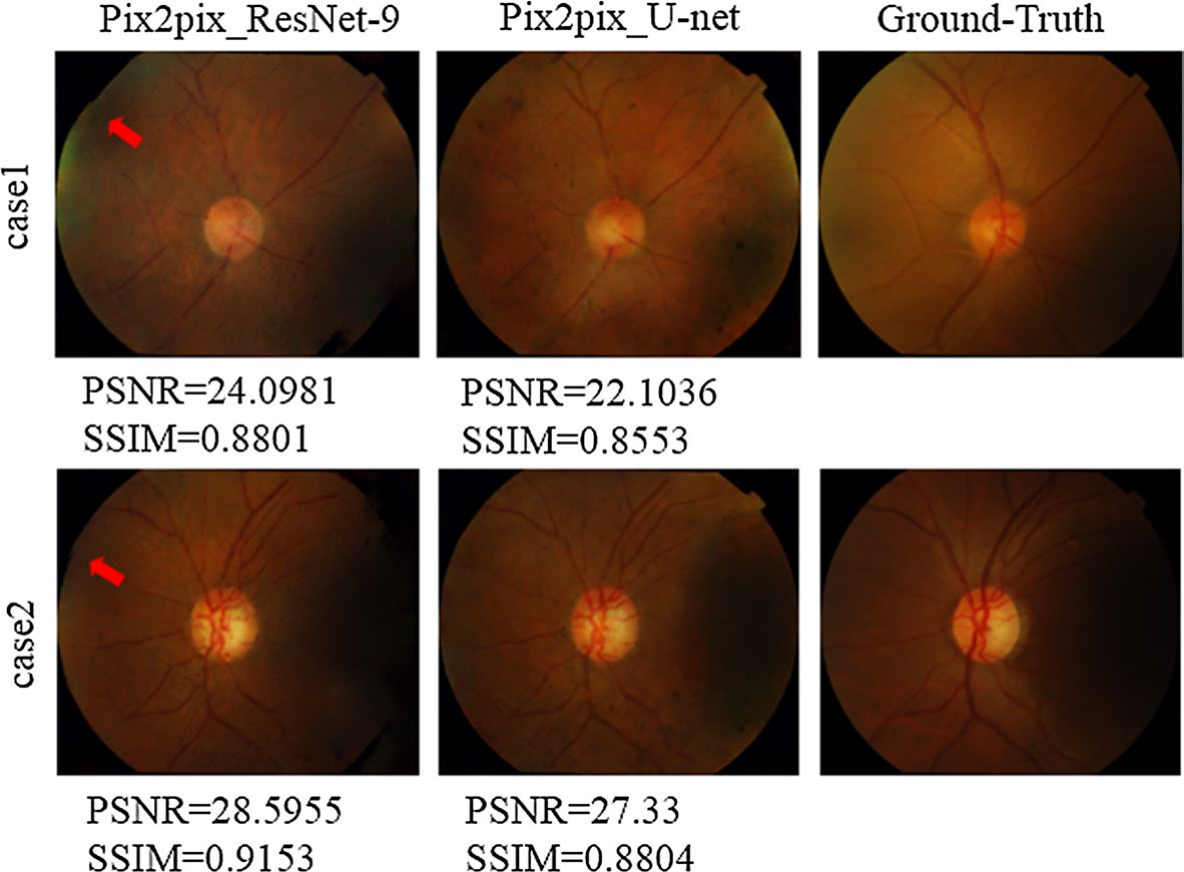
Special cases of synthetic results with ResNet-9 and U-net generator based on MCML method

Figure 5 demonstrates comparison studies using some selected test images for this task. It can be seen that U-net presents better quantitative performance and human visual perception than ResNet-9. The outline of the fundus image using ResNet-9 is not smooth and close to the circle. The unsmooth boundary of the images is marked by red rows. Given ResNet-9 presents better PSNR performance (Table 1), we have investigated MCML with different GANs on low-quality test images, which is presented in Fig. 6. As shown in the figures, since the quality of the test images is not good enough due to some dash area and uneven illumination, ResNet-9 has provided superior performance both PSNR and SSIM index better than U-net; however, U-net achieved better visual effect. This also explains sometimes why Single Vessel performs better than MCML, Cycle-GAN outperforms Pix2pix due to bad image quality at some cases. Nevertheless, we can conclude that U-net outperforms the ResNet-9 as the generator of the GANs in this work from the comprehensive comparison results.

#### Residual block analysis

Quantitative results for replacing different number of residual blocks are presented in Table 2. U-net with one-residual block has better performance than two or three blocks. The result shows that one-residual block combined with U-net can better solve the problem of useful information loss during convolution process, and more residual blocks stacking will produce another overfitting problem. Both the PSNR and SSIM of the one-residual-block U-net are better than the basic U-net in Table 1. Generally, optimized ResU-net is the best-performing network structure, which can be used as the generator of Pix2pix framework.

**Table 2:** Comparison of SSIM and PSNR with different number of residual blocks on U-net generator

	SSIM	PSNR
Residual-1	*0.9196*	*25.006*
Residual-2	0.9126	24.392
Residual-3	0.8941	23.375

Italic values are the best results

#### Resolution analysis

**Fig. 7:**
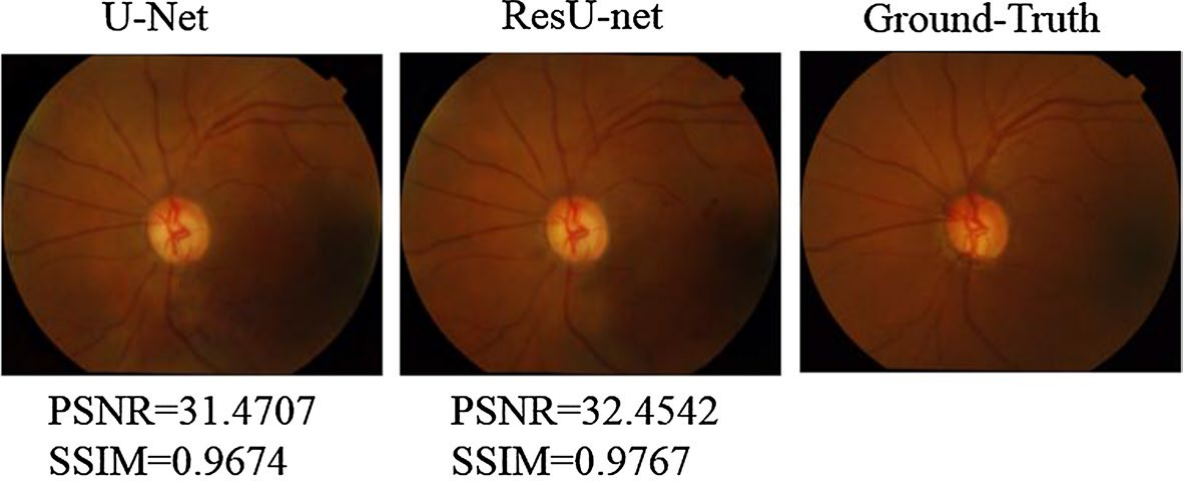
Synthetic result with U-net and ResU-net generator

In Costa et al. [20, 22], synthesized images were with a resolution of 256 × 256. They discussed the limitation of resolution that sometimes the synthetic fundus image with the same resolution is hard to distinguish veins from arteries. Therefore, we optimize the performance using an upsampling technique for further investigations. The images from DRISHTI-GS were upsampled to 512 × 512 as preprocessing, but afterwards corresponding synthetic results are downsampled to 256 × 256 by bicubic interpolation for quantitative evaluation. As shown in Fig. 7, both U-net generator and ResU-net generator achieved good visual effect. As presented in Table 3, the comparison of average SSIM and PSNR is listed. ResU-net generator achieved best PSNR and SSIM value with 512 × 512 images training.

**Table 3:** Comparison of SSIM and PSNR with different resolution image input and different improvement of generators

Generator	SSIM	PSNR
ResNet (256 × 256)	0.9079	25.3665
U-net (256 × 256)	0.9117	24.5543
U-net (512 × 512)	0.9208	25.0929
ResU-net (512 × 512)	*0.9361*	*25.7638*

Italic values are the best results

**Fig. 8:**
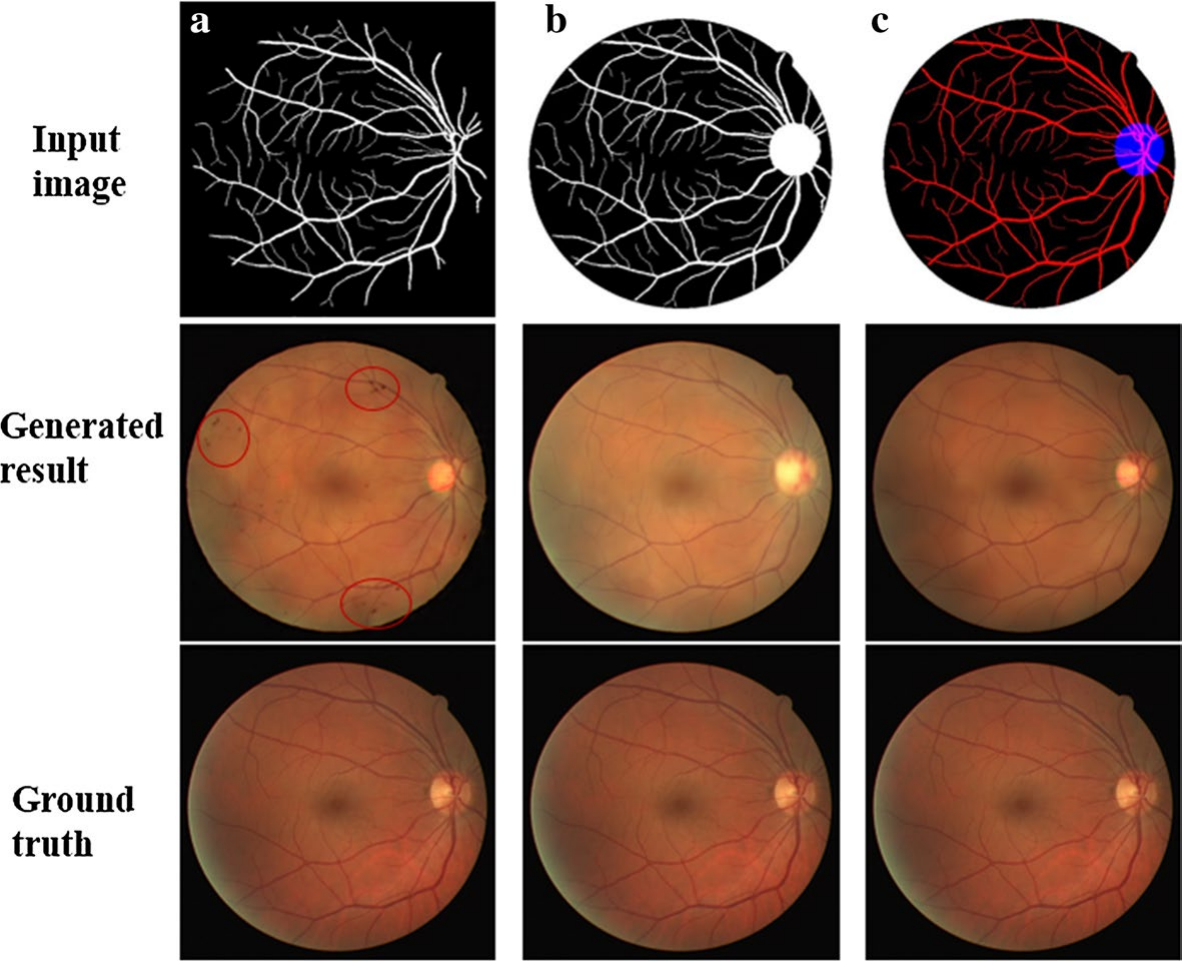
Synthetic results with different input methods. **a** Single vessel mask. **b** Optic disc and vessel fusion as one channel. **c** Optic disc and vessel fusion as multiple channels

In order to demonstrate the superiority of the MCML preprocessing pipeline, we have compared the best GAN model in this work with three different kinds of input methods on DRIVE dataset, which was used by all the prior works: (1) single vessel mask as input, (2) we combined optic disc with vessel as an image input, and (3) we use optic disc and vessel as two different channel inputs. As shown in Fig. 8, it can be seen that, the generated images based on MCML have achieved better visual perception than the other two methods.

We can observe that images generated from single vessel segmentation show vessel trees that are inconsistent and produce dark spots on the generated images, which are marked by the red circle in Fig. 8a. In addition to that, the optic disc and optic cup regions of generated images from single vessel input method are not clear. In Fig. 8b, since vessel tree, optic disc, and optic cup share an overlap area, if optic disc and vessel fusion are used as one channel, vessel and optic cup details are lost in the optic disc region. Finally, in Fig. 8c, the MCML method retains all the details of the different landmarks, generating better color and texture consistent results. From Table 4, we can see that the average PSNR and SSIM values of generated fundus image with MCML method outperform other methods.

**Table 4:** Comparison results of SSIM and PSNR with different landmarks input methods on DRIVE dataset

	SSIM	PSNR
Single vessel	0.9417	23.469
Fusion with one channel	0.9449	22.069
Fusion with multi-channel	*0.9498*	*23.722*

Italic values are the best results

## Discussion

In this paper, a multiple-channels-multiple-landmarks (MCML) GAN-based fundus image generation method was proposed. As discussed above, we can draw a conclusion that, the architecture of generator and the resolution of paired images, which are two essential properties of GANs, play key roles in generating high-quality image of synthetic fundus images.

Optic disc (OD) and optic cup (OC) are two important landmarks of the retina. The boundaries of the optic disc and the optic cup are essential for calculating the cup-to-disc ratio (CDR), which is an important indicator in detecting glaucoma [24]. As many glaucoma CAD tasks lack sufficient data, we think the MCML method can help researchers generate a large number of glaucoma images to improve their computer-aided diagnosis system. In prior fundus synthesis works, the generated images cannot achieve good results in optic disc region, whereas our MCML method can effectively solve this problem. According to our results, high-resolution images are suggested to use and then can leverage their own data to train again based on our pipeline. The advantage of the MCML method is that, the different sizes of optic disc and optic cup can be edited and revised, respectively, in the input mask, generating different kinds of realistic glaucoma fundus image with any CDR as required.

In addition, the MCML has potentials in the field of medical imaging field, such as pathology image, endoscope image, dermoscopy image, and other 2D medical images. Researchers can combine primary landmarks of these modality into multiple channels to generate better realistic medical images.

## Conclusions

In this work, we propose a multiple-channels-multiple-landmarks (MCML) method based on GANs to synthesize color fundus image from a combination of vessel, optic disc, and optic cup. The results demonstrate that our proposed method has superior performance compared to the fundus image generated from only vessel tree input. The synthesized images using the proposed method are realistic in look and contain more details, demonstrating better image quality. The MCML has many potentials in the medical imaging field, and can be applied into various lesions of fundus images generation and other medical image generation tasks as well. We will further investigate the relationship between the image quality of generated fundus image and the performance of computer-aided diagnosis system in future.

## Data Availability

The datasets used and/or analyzed during the current study are available from the corresponding author on reasonable request.
